# Advances in Research on *AARS1*/*AARS2*-Related Disorders: A Focus on Leukodystrophies

**DOI:** 10.3390/genes17080872

**Published:** 2026-07-26

**Authors:** Zhenyu Yang, Wendiao Zhang, Fengyi Yu, Xiaomei Duan, Miaojin Zhou, Beisha Tang

**Affiliations:** 1 Department of Neurology, Multi-Omics Research Center for Brain Disorders, The First Affiliated Hospital, Hengyang Medical School, University of South China, Hengyang 421001, China; 18774802508@163.com (Z.Y.); zhangwendiao@sklmg.edu.cn (W.Z.); drduanxm88@163.com (X.D.); 2 Clinical Research Center for Immune-Related Encephalopathy of Hunan Province, The First Affiliated Hospital, Hengyang Medical School, University of South China, Hengyang 421001, China; 3 Hengyang Medical School, University of South China, Hengyang 421001, China; yufengyi2016@email.szu.edu.cn (F.Y.); zhoumiaojin@usc.edu.cn (M.Z.); 4 The Key Laboratory of Pediatric Rare Diseases of the Ministry of Education, Hengyang and Central South University, University of South China, Changsha 410078, China; 5 Department of Neurology, Xiangya Hospital, Central South University, Changsha 410008, China; 6 National Clinical Research Center for Geriatric Disorders, Xiangya Hospital, Central South University, Changsha 410008, China

**Keywords:** leukodystrophies, alanyl tRNA synthetase, *AARS1*, *AARS2*

## Abstract

Leukodystrophies (LDs), a group of heterogeneous genetic disorders, are characterized by selective involvement of cerebral white matter, including abnormal white matter development and/or progressive degeneration. Oligodendrocytes, astrocytes, microglia, axons, and the neurovascular unit collectively contribute to white matter homeostasis and disease progression. Recently, genomic sequencing has identified pathogenic variants in the alanyl-tRNA synthetase 1 (*AARS1*) and alanyl-tRNA synthetase 2, mitochondrial (*AARS2*) genes in LD-related phenotypes. Dysfunction of AARS1 and AARS2 proteins may impair cytosolic or mitochondrial tRNA aminoacylation, compromise editing fidelity, and disrupt mitochondrial homeostasis, which may lead to disruption of protein homeostasis, cellular stress responses, and energy failure. Alanyl-tRNA synthetase (AlaRS) impairments play an important role in the pathological processes of cytosolic and mitochondrial alanyl-tRNA synthetase-related disorders. These molecular defects are associated with characteristic neuroimaging patterns and diverse clinical manifestations observed in *AARS1*/*AARS2*-related disorders. This review summarizes current knowledge on the genetic basis, clinicopathological features, and molecular mechanisms of *AARS1*- and *AARS2*-related leukodystrophies, and discusses emerging therapeutic perspectives, with the aim of facilitating precision diagnosis and future targeted interventions.

## 1. Introduction

Leukodystrophies (LDs), a group of genetic disorders, were first described by Bielschowsky in 1928 as hereditary diseases characterized by progressive degeneration of cerebral white matter [1]. Epidemiological data indicated an incidence ranging from approximately 1/50,000 to 1/7663; however, the true prevalence rate is likely underestimated due to disparities in diagnostic capability [2,3].

An increasing number of pathogenic genes associated with LDs have been identified through whole-exome sequencing and whole-genome sequencing. Classified by clinical phenotype and inheritance pattern, LDs encompass more than 30 distinct clinical phenotypes [4]. Notably, by adopting an expanded phenotypic method, up to 399 genes have been potentially associated with LDs and leukoencephalopathies; however, the majority of these genes still require further validation in clinical cases and functional experiments [4,5]. It is worth noting that the genes alanyl-tRNA synthetase 1 (*AARS1*; Online Mendelian Inheritance in Man [OMIM]: 601065) and alanyl-tRNA synthetase 2 (*AARS2*; OMIM: 612035), encoding cytosolic and mitochondrial alanyl-tRNA synthetase (AlaRS) proteins, were successively identified as pathogenic genes for LDs [6,7]. Therefore, this review focuses on *AARS1*- and *AARS2*-related LDs. In addition to summarizing their clinical and neuroimaging features, it systematically compiles the reported variants and reviews current evidence on genotype–phenotype relationships and disease mechanisms. It also evaluates the relevance and limitations of available experimental models and considers how current therapeutic evidence and experience from other LDs may inform the future development of therapeutic strategies for *AARS1*- and *AARS2*-related LDs.

## 2. *AARS1*/*AARS2*-Related Leukodystrophies

### 2.1. Leukodystrophy

In the 1980s, it was demonstrated that the core pathological mechanisms of LDs are closely associated with myelin abnormalities and oligodendroglial cell dysfunction [8]. As research progressed, evidence showed that the white matter microenvironment, rather than simply myelin injury, played a significant role in LDs, and comprised astrocytes, microglia, axons, and vascular units [8,9,10]. Based on these updated insights, LDs were classified into six major categories: 1. myelin disorders, which are primarily characterized by oligodendrocyte developmental defects or myelin metabolism abnormalities; these disorders were specifically divided into hypomyelinating disorders, demyelinating disorders, and myelin vacuolization diseases; 2. astrocytopathies, which are white matter disorders caused by astrocyte-specific genetic defects or functional dysregulation; 3. leuko-axonopathies, which are characterized by secondary white matter damage resulting from intrinsic neuronal or axonal defects and aberrant axon-glia interactions; 4. microgliopathies, which are characterized by white matter injury due to microglia-specific gene mutations or immune regulatory dysfunction; 5. leuko-vasculopathies, which are characterized by secondary white matter damage caused by primary genetic vascular pathological changes; and 6. other unclassified disorders [10].

The age at onset of LDs spanned all age groups, including childhood, adolescence, and adulthood. Notably, LDs exhibited heterogeneity in both clinical manifestations and genetic origins [2,4,9,10]. The clinical manifestations of LDs were complicated and diverse, comprising both neurological and non-neurological features. Neurological manifestations include: 1. neurodevelopmental disorders, such as delays in motor or language development; 2. progressive neurological dysfunction, including ataxia, cognitive decline, and epileptic seizures; 3. focal neurological deficits, such as stroke-like episodes and visual or auditory impairment; and 4. composite neurological signs, including pyramidal tract signs and peripheral neuropathy [2,11]. Non-neurological manifestations include: 1. ophthalmological symptoms, such as cataracts, optic nerve atrophy, and macular dystrophy; 2. endocrine dysfunction, including adrenal insufficiency and ovarian dysfunction; 3. polyneuropathy; 4. hypodontia; 5. cutaneous signs, such as xanthomas, ichthyosis, and acanthosis nigricans; and 6. visceral signs, including chronic diarrhea and gallbladder dysfunction [2].

Based on inheritance patterns, LDs were classified into the following categories: 1. autosomal dominant inheritance (AD), such as adult-onset autosomal dominant leukodystrophy (ADLD; OMIM: 621061) and Alexander disease (ALXDRD; OMIM: 203450); 2. autosomal recessive inheritance (AR), including globoid cell leukodystrophy (GLD, also known as Krabbe disease; OMIM: 245200) and progressive leukoencephalopathy with ovarian failure (LKENP; also referred to as *AARS2*-related leukoencephalopathy; OMIM: 615889); and 3. X-linked recessive inheritance (XLR), such as X-linked adrenoleukodystrophy (X-ALD; OMIM: 300100) [12].

### 2.2. AARS1-Related Leukodystrophy

The *AARS1* gene encodes the cytoplasmic enzyme AARS1, which belongs to the alanyl-tRNA synthetase family. This enzyme facilitated protein translation by catalyzing the covalent attachment of alanine to its cognate tRNA. Deleterious mutations in the *AARS1* gene impaired the aminoacylation activity of the encoded protein, which led to disease [6,13,14]. Such mutations resulted in an autosomal dominant form of hereditary diffuse leukoencephalopathy with spheroids-2 (HDLS2; OMIM: 619661), which is also referred to as adult-onset leukoencephalopathy with axonal spheroids and pigmented glia (ALSP) [6].

*AARS1*-related leukodystrophy typically had a relatively older age at onset, usually between 40 and 50 years of age; however, it was observed with an insidious onset and rapid progression [6]. There were few case reports of this subtype so far. Its clinical manifestations were characterized by sensorimotor impairment, cognitive decline, and behavioral abnormalities, which are usually accompanied by frontal lobe syndrome, pyramidal tract signs, dystonia, and hemianopia [6,13,14].

Neuroimaging features of *AARS1*-related leukodystrophy include symmetric hyperintense signals in the bilateral periventricular white matter on magnetic resonance imaging (MRI), particularly on fluid-attenuated inversion recovery (FLAIR) sequences, with involvement of the corpus callosum. Moreover, a progressive rim of decreased diffusion expanded centrifugally through the white matter from the periventricular area of the frontal and occipital horns [6,14]. Diffusion-weighted imaging (DWI) demonstrated restricted diffusion within white matter regions, which in progressive stages might affect the periventricular white matter and the splenium of the corpus callosum [13], potentially indicating axonal injury or myelin disruption. T1-weighted MRI revealed cortical atrophy, while subcortical U-fibers were relatively preserved in some patients. Computed tomography (CT) in a subset of patients showed symmetric patchy intracranial calcifications, most commonly reported in the basal ganglia, with possible involvement of the thalamus or cerebellar dentate nuclei (which were subtle or absent in early stages) [14].

Neuropathological analyses of *AARS1*-related leukodystrophy demonstrated typical pathological features of LDs, including myelin loss, axonal spheroid degeneration, and reactive gliosis in brain tissue [6,13,14].

In addition, pathogenic variants in the *AARS1* gene have been associated with several other inherited disorders, including autosomal dominant Charcot–Marie–Tooth disease type 2N (CMT2N; OMIM: 613287) [15], autosomal recessive developmental and epileptic encephalopathy type 29 (DEE29; OMIM: 616339) [16], and autosomal recessive non-photosensitive trichothiodystrophy type 8 (TTD8; OMIM: 619691) [17].

### 2.3. AARS2-Related Leukodystrophy

The *AARS2* gene encodes the mitochondrial alanyl-tRNA synthetase AARS2 enzyme [18,19]. It plays a critical role in mitochondrial protein translation by catalyzing the activity of the mitochondrial AARS2 enzyme and is also essential for mitochondrial energy metabolism and the respiratory chain [18,20]. Pathogenic variants in the *AARS2* gene have been shown to cause autosomal recessive ALSP or progressive leukodystrophy with ovarian failure [7,21].

*AARS2*-related leukodystrophy typically has an earlier age of onset, most commonly between 20 and 30 years of age [6,22], and fewer than 60 cases have been reported worldwide to date [23]. Its clinical phenotype is characterized by progressive motor dysfunction, including cerebellar ataxia and parkinsonism; motor-related speech and swallowing impairments, such as dysarthria and dysphagia; and psychiatric and behavioral abnormalities, including cognitive decline and changes in mood and personality [24,25]. Female patients additionally present with ovarian failure [22].

Neuroimaging features of *AARS2*-related leukodystrophy include the presence of peritrigonal white matter rarefaction with signal attenuation/suppression on MRI FLAIR sequences, along with mild thinning of the corpus callosum that was probably accompanied by hyperintensity. Diffusion-weighted imaging demonstrated punctate or partially confluent areas of restricted diffusion, most commonly involving the centrum semiovale, corona radiata, and the splenium of the corpus callosum. On T2-weighted MRI, asymmetric hyperintense signal changes predominantly affecting the frontoparietal white matter were observed, with asymmetric involvement of the corticospinal tracts extending into the posterior limb of the internal capsule and the brainstem [26,27]. CT typically shows no periventricular white matter calcifications [22].

A neuropathological analysis of *AARS2*-related leukodystrophy demonstrated myelin loss accompanied by white matter rarefaction and reactive gliosis. Numerous axonal spheroids were identified, exhibiting immunoreactivity for neurofilament proteins, p62, amyloid precursor protein (APP), and β-amyloid. Strongly scattered CD68-positive macrophages and microglia were observed within the lesions, indicating activation of phagocytic cells [24].

In addition, pathogenic variants in the *AARS2* gene have also been associated with autosomal recessive combined oxidative phosphorylation deficiency type 8 (COXPD8; OMIM: 614096) [20].

## 3. AARS1/AARS2: Protein Structure and Function

### 3.1. AARS1: Protein Structure and Function

**Figure 1:**
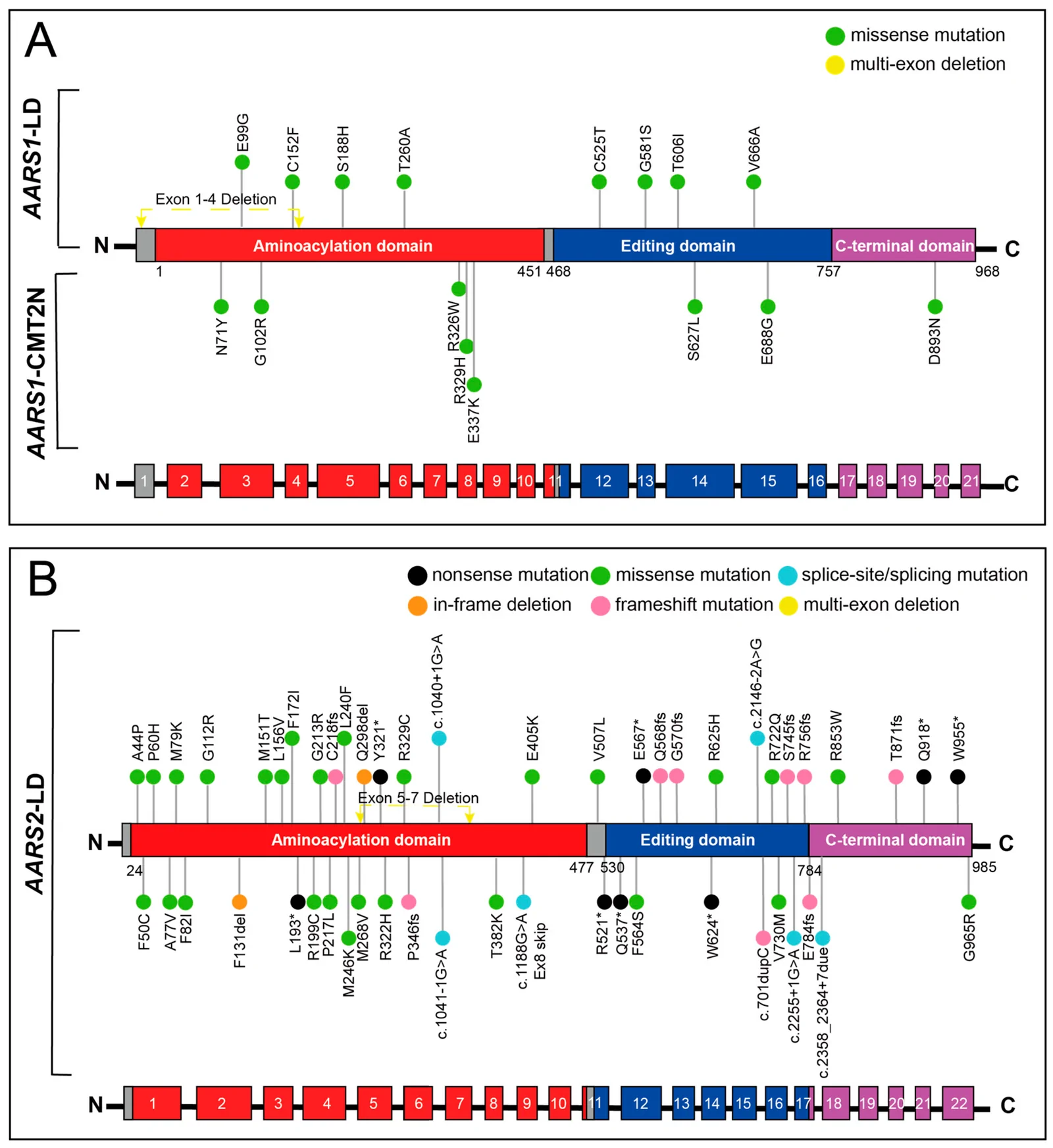
Schematic representation of the pathogenic variant spectrum of *AARS1* and *AARS2* across protein functional domains. (**A**) Distribution of reported *AARS1* variants associated with distinct clinical phenotypes, including leukodystrophy (LD) and Charcot-Marie-Tooth disease type 2N (CMT2N). Variants are mapped onto the aminoacylation domain (red), editing domain (blue), and C-terminal domain (purple) of *AARS1*. The yellow dashed bracket indicates the exon 1–4 deletion. Below the protein diagram, the corresponding exons are shown; the color of each exon matches the color of the protein domain it encodes (i.e., exons encoding the aminoacylation domain are red, those encoding the editing domain are blue, and those encoding the C-terminal domain are purple). (**B**) Distribution of reported *AARS2* variants associated with LD. Variants are mapped onto the aminoacylation, editing, and C-terminal domains of *AARS2*. The yellow dashed bracket indicates the exon 5–7 deletion. Splice-site/splicing variants, including canonical splice-site variants such as c.2146–2A>G and splice-affecting variants such as c.1188G>A leading to exon 8 skipping, are annotated according to HGVS nomenclature when applicable. Below the protein diagram, the corresponding exons are shown; the color of each exon matches the color of the protein domain it encodes. Green, black, cyan, orange, pink, and yellow dots indicate missense, nonsense, splice-site/splicing, in-frame deletion, frameshift, and multi-exon deletion mutations, respectively. The asterisk (*) indicates a premature stop codon. A comprehensive list of reported *AARS1* and *AARS2* variants and corresponding references is provided in Supplementary Tables S1 and S2.

The *AARS1* gene is located on human chromosome 16q22.1, contains 21 exons, and encodes the 968 amino acid cytoplasmic AARS1 protein. AARS1 belongs to the class II aminoacyl-tRNA synthetase (aaRS) family [28,29]. Structurally, the AARS1 protein contains three highly conserved functional domains arranged from the N-terminus to the C-terminus [30,31,32,33]. The aminoacylation domain, spanning residues 1–451, consists of a catalytic domain and a tRNA recognition domain [16]. The catalytic domain harbors the enzymatic active site responsible for catalyzing the aminoacylation reaction [30]. The tRNA recognition domain ensures specific recognition of alanyl-tRNA (tRNA^Ala^) through critical residues, especially the G3-U70 wobble base pair within the acceptor stem [30,34]. The editing domain, encompassing residues 468–757, mediates translational proofreading by recognizing and hydrolyzing incorrectly charged amino acids, such as glycine or serine misacylated onto cognate tRNAs [16,35]. The C-terminal domain, comprising residues 758–968, facilitates Ala-tRNA recognition and editing in prokaryotes [16,19,36]; however, in higher eukaryotes, including humans, evolutionary remodeling results in the loss of this canonical function and the acquisition of a novel role in nuclear DNA binding [37] (Figure 1A).

As a member of the aminoacyl-tRNA synthetase family, AARS1 possesses both canonical and non-canonical functions. Its canonical role is to keep accurate tRNA aminoacylation. Specifically, AARS1 catalyzes a two-step coupled reaction to ensure the correct loading of alanine onto its cognate tRNA^Ala^ during protein synthesis. Firstly, driven by adenosine triphosphate (ATP), alanine is activated to form alanyl-adenylate (Ala-AMP) with the concomitant release of pyrophosphate [35]. Subsequently, the activated alanine is transferred from Ala-AMP to the 3′ end of tRNA^Ala^ to generate alanyl-tRNA^Ala^ (Ala-tRNA^Ala^) [36]. Translational fidelity is maintained by strict substrate-specific recognition mechanisms. Finally, the generated Ala-tRNA^Ala^ is delivered to the ribosome for protein synthesis [38]. AARS1 develops a dual proofreading system to facilitate the accurate attachment of alanine to its cognate tRNA, in which incorrect aminoacyl-adenylate hydrolysis (e.g., Ser-AMP or Gly-AMP) is conducted by pre-transfer editing, while misacylated aminoacyl-tRNA elimination (e.g., Ser-tRNA^Ala^ or Gly-tRNA^Ala^) is conducted by post-transfer editing [39,40,41].

Non-canonical functions of aminoacyl-tRNA synthetases encompass a wide range of cellular activities, including the regulation of gene expression, RNA splicing, small-molecule metabolism, apoptosis and tumorigenesis, tissue development, angiogenesis, and immune responses [41,42]. As a member of this family, AARS1 also participates in several non-canonical cellular processes beyond its canonical aminoacylation activity.

In addition, AARS1 has been shown to function as a lactyltransferase independent of lactyl-coenzyme A, enabling it to directly sense intracellular lactate dynamics and catalyze protein lactylation [43].

### 3.2. AARS2: Protein Structure and Function

The *AARS2* gene is located on human chromosome 6p21.1, comprises 22 exons, and encodes the 985-amino acid mitochondrial AARS2 protein [23,44]. Similar to AARS1, AARS2 consists of three structural and functional domains. The aminoacylation domain, spanning residues 24–477 [45], contains a catalytic domain and a tRNA recognition domain. Unlike cytosolic tRNA^Ala^, mitochondrial tRNA^Ala^ lacks the conserved G3-U70 base pair. Instead, mitochondrial tRNA^Ala^ recognition is mediated by the A1-U72, A2-U71, and G4-C69 base pairs, the discriminator base A73, as well as the G5-U68 wobble base pair, which contribute to recognition efficiency [35,36,38]. The editing domain encompasses residues 530–783, whereas the C-terminal domain spans residues 784–985 [45] (Figure 1B).

Similar to AARS1, AARS2 functions as an alanyl-tRNA synthetase that catalyzes the accurate conjugation of alanine to its cognate mitochondrial tRNA through a two-step coupled reaction, thereby providing an essential substrate for mitochondrial protein synthesis; in addition, AARS2 has also been implicated in non-canonical functions, including participation in protein lactylation and related cellular processes [46].

## 4. Current Status and Pathomechanisms of *AARS1*/*AARS2*-Related Leukodystrophies

### 4.1. Mutational Spectrum Characteristics of AARS1-Related Leukodystrophy

*AARS1*-related leukodystrophy was first reported in 2019, when two affected individuals from a single HDLS family were identified as heterozygous c.455G>T (p.Cys152Phe) *AARS1* gene variant carriers. Localized to the aminoacylation domain, this variant was predicted to be potentially pathogenic and to impair protein function [6]. In 2021, among 11 carriers of biallelic *AARS1* variants, four individuals were found to exhibit white matter abnormalities, and seven variants were identified, including compound heterozygous variants c.296A>G(p.Glu99Gly) with c.778A>G(p.Thr260Ala), c.562_563delinsCA(p.Ser188His) with c.1574G>A(p.Cys525Tyr), the homozygous variant c.1741G>A(p.Gly581Ser), etc. [13]. These variants were distributed across the aminoacylation and editing domains of AARS1. In 2023, an autosomal recessive adult-onset leukoencephalopathy with axonal spheroids and pigmented glia patient was reported to carry a homozygous c.1817C>T(p.Thr606Ile) variant located in the editing domain, which markedly reduced enzymatic catalytic activity and significantly increased the rate of amino acid mischarging [14]. To date, a limited number of genetically confirmed *AARS1*-related leukodystrophy cases have been reported worldwide, with nine distinct pathogenic variants identified (Figure 1A and Supplementary Tables S1 and S2) [6,13,14].

Apart from LDs, *AARS1* mutations have also been implicated in peripheral neuropathies, such as Charcot–Marie–Tooth disease type 2N. *AARS1* mutations show different characteristics between the central nervous system and the peripheral nervous system.

*AARS1*-related Charcot–Marie–Tooth disease type 2N was first reported in 2010, when 18 carriers from two CMT2 families were identified as carrying a heterozygous *AARS1* variant c.986G>A(p.Arg329His), which was located within the aminoacylation domain and was observed to be potentially pathogenic [34]. Charcot–Marie–Tooth disease is the most common inherited neuromuscular disorder, with an estimated global prevalence of approximately 1 in 2500 [34]. Typical clinical manifestations of CMT include progressive distal muscle weakness, sensory impairment, and reduced tendon reflexes [47,48]. Based on electrophysiological and pathological characteristics, CMT was broadly classified into demyelinating forms (CMT1) and axonal forms (CMT2), and intermediate forms [49]. CMT2N caused by *AARS1* mutations is characterized by adult-onset distal axonal peripheral neuropathy, presenting with progressive muscle weakness, sensory loss, and diminished tendon reflexes. Moreover, foot deformities were also observed. Electrophysiological findings predominantly indicated axonal damage, with no evidence of central nervous system damage [50,51], which differed from *AARS1*-related LDs. To date, *AARS1*-related CMT2N has been reported in multiple families, encompassing a total of eight variants (Figure 1A and Supplementary Table S1) [15,31,34,50,52,53,54], most of which were located within the catalytic domain, with a smaller proportion distributed in the editing and C-terminal domains [50,55]. Notably, the c.986G>A(p.Arg329His) variant has been repeatedly identified across different ethnic populations [34,51,56], suggesting that this site may represent a mutational hotspot.

### 4.2. Mutational Spectrum Characteristics of AARS2-Related Leukodystrophy

*AARS2*-related leukodystrophy was first reported in 2014, when 11 distinct *AARS2* variants were identified in six patients with unexplained white matter abnormalities, including six missense variants, four nonsense/frameshift variants, and one in-frame deletion. These variants were distributed across the aminoacylation domain (6/11), the editing domain (5/11), and comprised compound heterozygous combinations such as c.149T>G(p.Phe50Cys) with c.1561C>T(p.Arg521*), and c.2893G>A(p.Gly965Arg) with c.1213G>A(p.Glu405Lys). The pathogenicity of only the c.149T>G(p.Phe50Cys) and c.1561C>T(p.Arg521*) variants was demonstrated in a yeast model in vitro, whereas the remaining variants were predicted by in silico analyses [7]. To date, a total of 55 patients with *AARS2*-related leukodystrophy have been reported, encompassing 53 distinct variants, among which c.1871G>A(p.Trp624*) and c.452T> C(p.Met151Thr) may represent recurrent hotspot mutations (Figure 1B and Supplementary Tables S1 and S2) [7,23,24,25,57,58,59,60,61,62,63,64,65,66,67,68,69,70,71,72,73,74,75,76,77,78,79,80,81,82,83,84,85,86].

### 4.3. Pathomechanisms of AARS1/AARS2-Related Disorders

At present, the pathomechanisms of *AARS1*/*AARS2*-related leukodystrophies have not been fully elucidated. Insights have been partially derived from other *AARS1*/*AARS2*-related disorders, such as CMT2N, DEE29, and COXPD8, as well as from the biological functions of AARS1/AARS2 proteins. Collectively, current evidence primarily supports disruption of the canonical aminoacylation and editing functions of *AARS1* and *AARS2*, whereas mutation-specific alterations in non-canonical functions may contribute in selected contexts; the relevance of AARS1/AARS2-mediated protein lactylation to leukodystrophy remains uncertain.

**Figure 2:**
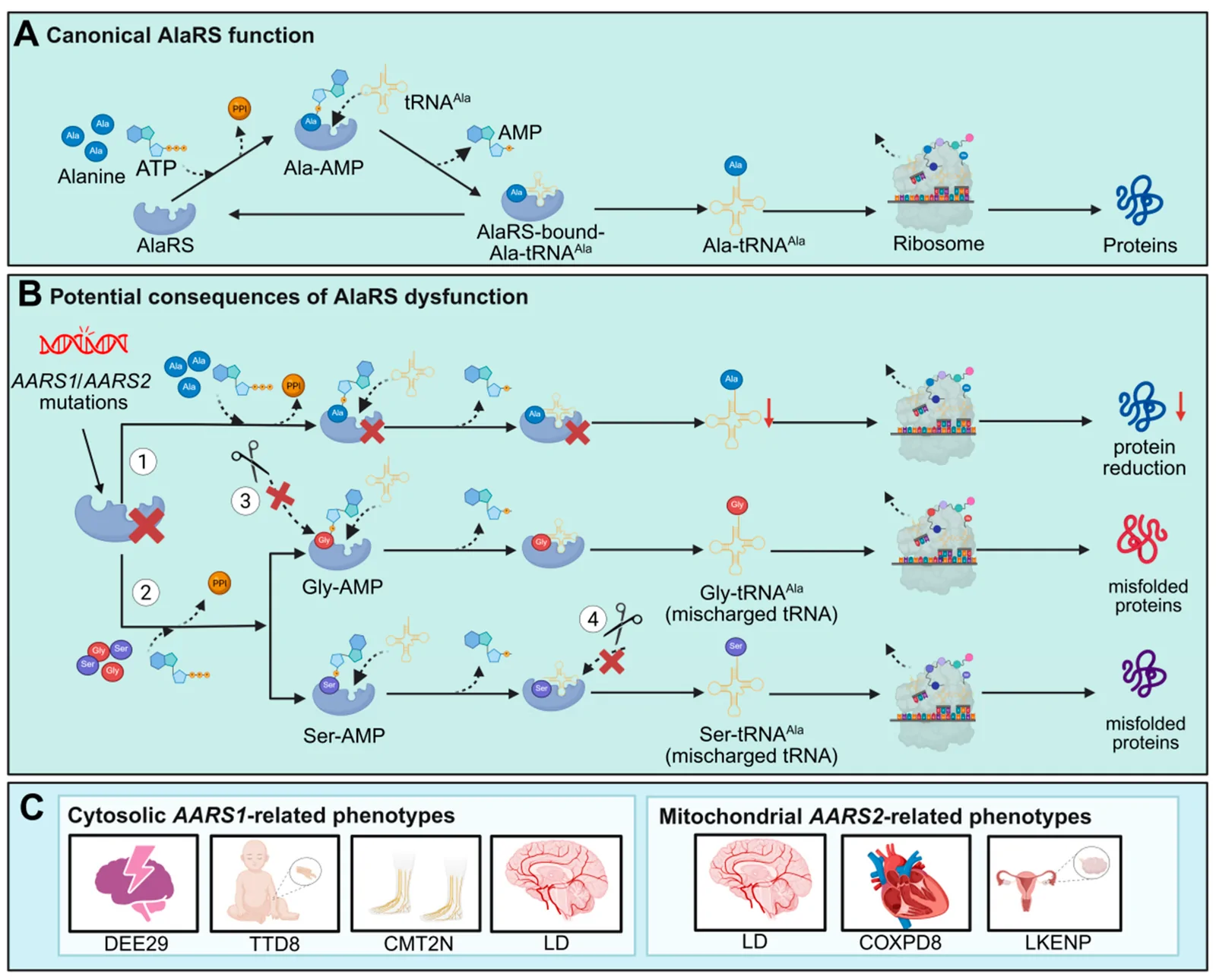
Canonical function and potential pathogenic consequences of AlaRS dysfunction. (**A**) AlaRS catalyzes the aminoacylation of tRNA^Ala^ by first activating alanine with ATP to form Ala-AMP, followed by transfer of alanine to tRNA^Ala^ to generate AlaRS-bound Ala-tRNA^Ala^ and released Ala-tRNA^Ala^ for ribosomal protein synthesis. (**B**) AlaRS dysfunction may reduce Ala-tRNA^Ala^ formation and impair protein synthesis. Defective substrate discrimination or editing may also permit misactivation of non-cognate amino acids, such as glycine or serine, leading to mischarged Gly-tRNA^Ala^ or Ser-tRNA^Ala^, mistranslation, and protein misfolding. Numbers indicate representative steps affected by AlaRS dysfunction: ① impaired AlaRS activity; ② non-cognate amino acid misactivation; ③ defective pre-transfer editing; and ④ defective post-transfer editing. Solid black arrows indicate the direction of reaction flow and pathway progression, whereas dashed black arrows indicate substrate input or product release. Red downward arrows indicate reduced abundance or output, and red crosses indicate AlaRS dysfunction or impairment of the corresponding catalytic or editing step. Scissor symbols represent the editing or proofreading function of AlaRS. (**C**) Representative phenotypes associated with cytosolic *AARS1* and mitochondrial *AARS2* variants. *AARS1* variants have been associated with developmental and epileptic encephalopathy type 29 (DEE29), trichothiodystrophy type 8 (TTD8), Charcot–Marie–Tooth disease type 2N (CMT2N), and leukodystrophy (LD). *AARS2* variants have been associated with LD, combined oxidative phosphorylation deficiency type 8 (COXPD8), and leukoencephalopathy with ovarian failure (LKENP). Abbreviations: AlaRS, alanyl-tRNA synthetase; *AARS1*, alanyl-tRNA synthetase 1; *AARS2*, alanyl-tRNA synthetase 2, mitochondrial; Ala, alanine; ATP, adenosine triphosphate; AMP, adenosine monophosphate; PPi, inorganic pyrophosphate; Ala-AMP, alanyl-adenylate; tRNA^Ala^, alanine transfer RNA; Ala-tRNA^Ala^, alanyl-tRNA^Ala^; Gly-tRNA^Ala^, glycyl-tRNA^Ala^; Ser-tRNA^Ala^, seryl-tRNA^Ala^. Created in BioRender. Fengyi, Y. (2026) https://BioRender.com/reobrzi (accessed on 7 June 2026).

Defects in the canonical function primarily manifest as reduced aminoacylation activity or compromised editing capacity, thereby impairing accurate tRNA charging and translational fidelity [19,30]. Pathogenic variants of *AARS1*/*AARS2* interfere with accurate protein synthesis or promote the accumulation of mistranslated or misfolded proteins. These alterations are associated with neuronal cell death [87], cardiomyocyte apoptosis [44,45], and ATP production defects in transfected ovarian granulosa tumor cells [88], which is consistent with disease-relevant cellular phenotypes underlying neurological manifestations, cardiomyopathy, and premature ovarian failure (Figure 2). High-energy-demand tissues, such as the central nervous system and skeletal muscle, may be particularly vulnerable to ATP insufficiency and AARS1/AARS2 dysfunction, potentially contributing to white matter injury and dysfunction [41,87].

Alongside the bioenergetic consequences described above, impaired translational fidelity and the resulting accumulation of misfolded proteins may activate proteotoxic stress responses. In the *aars1* zebrafish model, ubiquitinated protein accumulation is accompanied by endoplasmic reticulum stress and protein kinase R-like endoplasmic reticulum kinase (PERK) signaling [89], whereas in the homozygous *Aars1^sti/sti^* mouse model, the accumulation of misfolded and ubiquitinated proteins, endoplasmic reticulum stress, and activation of the unfolded protein response (UPR) have been shown [19,87]. These findings support UPR-related signaling as a potential downstream consequence of AARS1 dysfunction. However, leukodystrophy-associated white matter pathology has not been demonstrated in either model, and the relevance of this mechanism to human *AARS1*/*AARS2*-related leukodystrophies remains uncertain. In a study on patient-derived induced neural progenitor cells (iNPCs) carrying *AARS2* variants, transcriptomic enrichment of heme-regulated inhibitor (HRI)-related integrated stress response (ISR) signaling was observed, whereas increased eukaryotic initiation factor 2α (eIF2α) phosphorylation was not detected [90]. Because HRI-related ISR signaling does not itself establish activation of the canonical endoplasmic reticulum UPR, direct evidence for UPR activation in *AARS2*-related leukodystrophy remains limited.

Beyond their canonical aminoacylation functions, aminoacyl-tRNA synthetases also participate in diverse non-canonical cellular processes [91]. In the context of *AARS1*-associated disease, a mutation-specific non-canonical mechanism has been proposed for CMT2N. The *AARS1* mutation c.986G>A(p.Arg329His), located within the aminoacylation domain, did not measurably impair tRNA aminoacylation in experiments using either purified protein or patient-derived cells. Instead, the mutant protein acquired the ability to interact aberrantly with neuropilin-1 (Nrp1). This interaction was also confirmed in patient-derived lymphocytes carrying the mutation and was proposed as a potential gain-of-function mechanism in CMT2N. By contrast, the editing-domain and C-Ala-domain mutations examined in the same study did not induce this interaction [30].

More recently, AARS1 and AARS2 have been reported to act as intracellular L-lactate sensors and protein lysine lactyltransferases in several experimental contexts [46,92,93]. Purified protein assays further showed that AARS1/AARS2-mediated lactylation was dependent on L-lactate and ATP but did not require lactate dehydrogenase (LDH) in vitro [46,93]. In tumor cell models, AARS1-mediated lactylation of p53 impaired its liquid–liquid phase separation, DNA binding, and transcriptional activity [92]. In immune-related cellular and mouse models, AARS2 was associated with cyclic GMP–AMP synthase (cGAS) in the cytoplasm and mediated its lactylation and inactivation, thereby suppressing the cGAS–stimulator of interferon genes (STING) signaling pathway [46]. In mouse muscle cells and skeletal muscle in vivo, AARS2-mediated lactylation of pyruvate dehydrogenase E1 alpha subunit (PDHA1) and carnitine palmitoyltransferase 2 (CPT2) reduced their enzymatic activities and limited oxidative phosphorylation under hypoxic conditions [93]. Although lactate was detected in isolated mitochondrial fractions and inhibition of monocarboxylate transport was associated with reductions in both lactate within these fractions and the lactylation of PDHA1 and CPT2 [93], the physiological sources of the mitochondrial lactate pool and the precise route by which lactate becomes available to mitochondrial AARS2, particularly in human neural cells, remain unresolved.

Given that ovarian failure is an important clinical feature among women with *AARS2*-related leukodystrophy [7], a subsequent study using a human granulosa cell line and mouse models supported a model in which AARS2-dependent lactylation and inactivation of CPT2 and PDHA1 promoted follicular development and subsequent follicular depletion [94]. Several *AARS2* variants that were classified as primary ovarian insufficiency-associated in that study displayed increased lactyltransferase activity [94], suggesting a possible contribution of increased AARS2-mediated lactylation to ovarian dysfunction. However, these findings were derived from non-neural experimental systems, and no direct evidence currently links AARS1/AARS2-mediated lactylation to the neurological or white matter manifestations of these disorders. The contribution of lactylation to leukodystrophy therefore remains hypothetical.

Defects in tRNA aminoacylation or editing are common across aminoacyl-tRNA synthetase (aaRS)-related disorders, but downstream responses differ. Patient-derived induced iNPC lines carrying variants in *AARS2*, mitochondrial glutamyl-tRNA synthetase 2 (*EARS2*), or mitochondrial arginyl-tRNA synthetase 2 (*RARS2*) all showed impaired mitochondrial respiration. The *AARS2* and *EARS2* lines had lower ATP production and relied more on glycolysis. The *RARS2* line instead showed increased proton leak and activation of general control nonderepressible 2 (GCN2)–eIF2α signaling without reduced cytosolic translation [90]. Thus, mitochondrial aaRS defects may share bioenergetic dysfunction but differ in their compensatory responses. Non-canonical effects appear more variant-specific. In CMT2N, the AARS1 p.Arg329His mutant protein exhibited aberrant binding to Nrp1 [30]. In CMT2D, several mutant forms of glycyl-tRNA synthetase 1 (GARS1) also bound Nrp1, although the strength of this interaction differed among the variants examined [95]. Abnormal protein interactions may therefore recur in some aaRS-associated peripheral neuropathies, while their downstream effects remain enzyme- and variant-dependent. Among the aaRSs examined to date, direct L-lactate- and ATP-dependent protein lysine lactylation has been demonstrated only for AARS1, AARS2, and bacterial alanyl-tRNA synthetase (AlaRS) [46,92,93]. Thus, this activity is a distinctive non-canonical function of AARS1/AARS2. However, no direct evidence yet links it to *AARS1*/*AARS2*-related leukodystrophies.

### 4.4. Experimental Models Relevant to AARS1/AARS2-Related Disorders

Experimental models provide important insights into the molecular consequences of pathogenic variants in *AARS1* and *AARS2*. The available systems include humanized yeast, zebrafish, and mouse models, and patient-derived human induced pluripotent stem cells (hiPSCs), iNPCs, and neurons. Most of these models are designed to investigate specific variants or phenotypes rather than to fully recapitulate the neuropathology of *AARS1*/*AARS2*-related leukodystrophies. The following discussion critically compares their strengths, limitations, and relevance to these disorders.

At the reductionist level, a humanized *Saccharomyces cerevisiae* model was used to test 16 recessive-disease-associated *AARS1* missense variants, of which 11 showed partial or complete loss-of-function effects, while c.242A>C(p.Lys81Thr) and c.296A>G(p.Glu99Gly) also exerted dominant-negative effects [96]. Although suitable for rapid variant screening, this model may yield false-negative results and cannot reliably evaluate genotype–phenotype relationships or recapitulate human neural and white matter pathology [96].

Moving from reductionist assays to vertebrate models, zebrafish provide a rapid in vivo system for examining the effects of *aars1* loss of function on embryonic neurogenesis. The *aars1^cq71^*^/*cq71*^ null mutant showed ubiquitinated protein accumulation, endoplasmic reticulum stress, Perk–Nfe2l2b–p53 axis-dependent apoptosis of neural progenitor cells, and impaired neurogenesis [89]. This model enables rapid in vivo analysis of neural development and rescue effects; however, this null model does not carry a patient-specific variant, and leukodystrophy-associated white matter pathology has not been assessed.

Mouse models provide a complementary mammalian context for investigating the in vivo consequences of *AARS1*/*AARS2* variants. In the *Aars1^sti/sti^* mouse model, the editing-domain missense mutation c.2201C>A(p.Ala734Glu) impaired the proofreading activity of the AARS1 enzyme and led to serine mischarging of tRNA^Ala^. In these *Aars1* mutant mice, translational infidelity subsequently resulted in aberrant accumulation of misfolded and ubiquitinated proteins within neurons, which was accompanied by endoplasmic reticulum stress and activation of the unfolded protein response, ultimately leading to the progressive apoptotic loss of cerebellar Purkinje cells. Consequently, these *Aars1* mutant mice exhibited progressive ataxia. In addition, they exhibited coat abnormalities, including rough, unkempt fur, follicular dystrophy, and patchy hair loss [19,87]. Regarding *Aars2*, homozygous knock-in mouse models harboring editing-domain mutations c.2230TG>GC(p.Cys744Ala) and c.2264T>A(p.Val755Glu) demonstrated that loss of AARS2 proofreading activity resulted in tRNA mischarging and translational infidelity, ultimately leading to early embryonic lethality. In contrast, heterozygous mice showed no overt abnormalities, possibly because the wild-type AARS2 enzyme compensated for the editing defect through trans-editing. Compared with the late-onset cerebellar degeneration observed in the *Aars1^sti/sti^* mouse model, the early embryonic lethality associated with homozygous AARS2 editing defects has been proposed to reflect the high sensitivity of mitochondrial translation to serine misincorporation and the absence of the free-standing editing-domain homolog AlaXp, also known as alanyl-tRNA synthetase domain-containing protein 1 (AARSD1), within mitochondria [97].

Together, these mouse models enable the in vivo investigation of the effects of impaired translational proofreading on protein homeostasis, neuronal vulnerability, and embryonic development. However, these models primarily represent editing-domain dysfunction. The *Aars1^sti/sti^* mouse predominantly recapitulates cerebellar degeneration, whereas embryonic lethality in the homozygous *Aars2* mutant mice precludes the investigation of postnatal neurological progression. Moreover, these mouse models have not been shown to recapitulate the characteristic white matter pathology of *AARS1*/*AARS2*-related leukodystrophies, limiting their direct translational relevance to these disorders.

Complementing these animal models, patient-derived cellular systems provide a human genetic context for investigating the cellular consequences of pathogenic variants in *AARS1* and *AARS2* dysfunction. For *AARS1*, fibroblasts from a patient with CMT2N carrying the heterozygous c.986G>A(p.Arg329His) variant were reprogrammed into hiPSCs, and CRISPR-Cas9-corrected isogenic clones retaining pluripotency were generated. However, these cells were not differentiated into neural lineages or characterized for disease-related phenotypes and therefore represent a cellular resource rather than a functional model of *AARS1*-related leukodystrophy [98]. For *AARS2*, one study reprogrammed fibroblasts derived from patients with *AARS2*-associated COXPD8 into hiPSCs and validated their pluripotency. This work primarily aimed to expand human hiPSC resources for *AARS2*-related disorders and did not include further functional experiments addressing disease mechanisms [99]. Thus, these hiPSC lines provide a renewable resource for future studies rather than a functionally characterized model of neural or white matter pathology.

In a separate study, fibroblasts from a patient with *AARS2*-related leukodystrophy were directly converted into iNPCs, which were further differentiated into neurons. The results showed that *AARS2* deficiency is associated with defective mitochondrial protein translation in neurons, accompanied by reduced levels of subunits of respiratory chain complexes I, III, and IV, impaired oxidative phosphorylation, and a metabolic shift toward increased glycolytic dependence. Transcriptomic analyses of the *AARS2* iNPCs further indicated downregulation of neurodevelopment-related pathways, including axon guidance, transforming growth factor-β (TGF-β), and mitogen-activated protein kinase (MAPK) signaling, together with widespread disturbances in RNA regulatory processes. In addition, enrichment of heme-regulated inhibitor (HRI)-related integrated stress response (ISR) signaling was observed in these cells, although increased phosphorylation of eIF2α was not detected [90]. Collectively, these findings indicated that pathogenic variants in *AARS2* were associated with mitochondrial translational defects and impaired neuronal development.

Together, these cellular models retain patient-specific genetic backgrounds but differ in their experimental maturity and relevance to leukodystrophy. The hiPSC lines carrying *AARS1* and *AARS2* variants offer self-renewal, scalability, and differentiation potential; however, they were derived from patients with CMT2N and COXPD8, respectively, and neither has undergone neural differentiation or disease-related functional characterization. Their relevance to leukodystrophy therefore remains indirect, although the paired parental and gene- *AARS1*-corrected lines provide an isogenic platform for future mechanistic studies. By contrast, the iNPC-derived neurons carrying *AARS2* variants capture mitochondrial translational and metabolic abnormalities associated with leukodystrophy, but the available evidence remains limited, and the study lacked an isogenic gene-corrected control. Moreover, none of these cellular models address oligodendrocyte differentiation, myelination, or multicellular white matter architecture. Patient-derived neural models of *AARS1*-related leukodystrophy and multicellular models of both disorders therefore remain lacking.

Across experimental systems, existing models reproduce selected molecular and cellular consequences of pathogenic variants in *AARS1* and *AARS2*, but none have been shown to recapitulate the multicellular white matter pathology or temporal progression of the human disorders. Mechanistic evidence from animal models remains concentrated on null or editing-domain variants, whereas functional studies in patient-derived neural cells remain limited. These gaps restrict the evaluation of variant-specific mechanisms and genotype–phenotype relationships.

Because *AARS1*/*AARS2*-related leukodystrophies encompass both early-onset neurodevelopmental abnormalities and adult-onset progressive neurodegeneration, human brain organoids may provide a useful complement to existing models. However, to the best of our knowledge, no *AARS1*/*AARS2*-specific brain organoid model has yet been reported. Patient-derived myelinating brain organoids, ideally paired with isogenic gene-corrected controls, could enable three-dimensional investigation of neural–glial interactions, oligodendrocyte development, and myelination [100,101,102]. Nevertheless, their predominantly fetal or early postnatal maturation state, protocol-dependent variability, and the frequent absence of functional vasculature and microglia in conventional protocols may restrict their ability to reproduce adult-onset disease progression; long-term maintenance of mature myelin may also remain challenging in some systems [103]. Integration of organoids with established two-dimensional cellular and animal models will therefore be necessary for cross-model validation of disease mechanisms and potential therapeutic targets.

## 5. Genotype–Phenotype Correlations of *AARS1*/*AARS2*

*AARS1* mutations are predominantly located within the aminoacylation or editing domains, leading to impaired aminoacylation, defective editing activity, and protein instability. These abnormalities gave rise to diverse clinical phenotypes, including LDs, distal sensorimotor neuropathy (CMT2N), and developmental and epileptic encephalopathy (DEE29). For example, the heterozygous variant c.455G>T(p.Cys152Phe) in the aminoacylation domain and the homozygous variant c.1817C>T(p.Thr606Ile) in the editing domain have been associated with adult-onset leukodystrophy, which is characterized by cognitive decline, motor dysfunction, and white matter demyelination, with a median survival typically of less than 10 years [6,14]. A heterozygous variant c.986G>A(p.Arg329His) in the aminoacylation domain has been associated with CMT2N, presenting as distal sensorimotor neuropathy with progressive muscle weakness, sensory loss, and axonal peripheral neuropathy [47,55]. By contrast, compound heterozygous variants involving c.242A>C(p.Lys81Thr) in the aminoacylation domain and c.2251A>G(p.Arg751Gly) in the editing domain, or c.2067dupC (p.Tyr690Leufs*3) in the editing domain combined with c.2738G>A(p.Gly913Asp) in the C-terminal domain, have been associated with DEE29, manifesting as severe infantile epileptic encephalopathy accompanied by hypomyelination and peripheral neuropathy [16]. However, a clear genotype–phenotype correlation for the *AARS1* gene has not yet been established [6].

Genotype–phenotype correlations of the *AARS2* gene show a certain association with mutation sites and the extent of specific domain functional impairments [104]. The compound heterozygous variants c.149T>G(p.Phe50Cys) and c.1561C>T(p.Arg521*) have been located in the aminoacylation domain [30]. As a result, these mutations are related to adult-onset leukodystrophy and characterized by progressive white matter degeneration, cognitive impairment, and cerebellar ataxia, with female patients frequently accompanied by premature ovarian failure [34]. In another study, homozygous or compound heterozygous variants involving c.1774C>T(p.Arg592Trp) located in the editing domain disrupted AARS2 editing activity and led to combined oxidative phosphorylation deficiency type 8, which manifested as infantile cardiomegaly, progressive heart failure, respiratory failure, and early death. Laboratory investigations revealed severe deficiencies of mitochondrial respiratory chain complexes I and IV, accompanied by lactic acidosis [44]. In addition, compound heterozygous variants involving c.1738C>T(p.Arg580Trp) in the editing domain and c.1008dupT (p.Asp337*) in the aminoacylation domain have been shown to impair protein stability and induce a lethal phenotype, with marked reductions in respiratory chain complex activities and abnormal lactate accumulation [105].

At the time of writing, it was generally accepted that *AARS2* mutation locations and the extent of domain-specific functional impairment are closely associated with clinical phenotypes.

## 6. Treatment of *AARS1*/*AARS2*-Related Leukodystrophies

Clinical management of *AARS1*/*AARS2*-related leukodystrophy is generally based on principles of early diagnosis, multidisciplinary collaboration, integrated intervention, and long-term care, aiming to improve quality of life, reduce complications, and prolong survival. At present, treatment remains primarily supportive and symptomatic, with no disease-specific therapy available to reverse or substantially slow disease progression. Therefore, therapeutic approaches explored in other LDs are considered informative for clinical management.

### 6.1. Symptomatic and Supportive Pharmacological Treatment

Symptomatic medications are selected according to the patient’s clinical manifestations. These include antiparkinsonian agents for movement disorders [106], cholinesterase inhibitors for cognitive impairment [26], antidepressants and antipsychotics for affective and psychiatric symptoms [24,106], antiepileptic drugs for seizure control [7], muscle relaxants for abnormal muscle tone [13], and coenzyme supplements [104]. The therapeutic effects of coenzyme supplementation in *AARS2*-related leukodystrophy are typically transient, with frequent relapse after discontinuation, indicating limited long-term benefit [64]. In addition, amino acid supplementation has been explored as a supportive metabolic approach for mitochondrial aminoacyl-tRNA synthetase-related leukodystrophies. In a small pilot study of *AARS2*- and mitochondrial aspartyl-tRNA synthetase *(DARS2)*-related leukodystrophies, supplementation was reported to be generally safe in the overall cohort. Among the two adult patients with *AARS2*-related disease who received alanine, no clear clinical benefit was observed. One patient completed the trial. The other withdrew early because of rapid cognitive deterioration, but no causal relationship with alanine therapy could be established [84].

### 6.2. Rehabilitation Training and Care

The combination of gait training, functional rehabilitation, medications, and supportive therapies is associated with improvements in motor function and basic activities of daily living, such as bathing and eating. However, limited improvements in complex instrumental activities of daily living have been observed. Notably, in patients with rapidly progressive disease, the benefits of rehabilitation and care interventions are often unsatisfactory [73].

### 6.3. Immunosuppressive Therapy

Evidence indicates that immunosuppressive agents, particularly glucocorticoids, provide only limited and transient symptomatic benefit in *AARS2*-related leukodystrophy [62]. Some patients showed no appreciable response to immunosuppressive agents [25]. These findings suggest that immunosuppression is not an effective therapeutic strategy, and its clinical value requires validation through longitudinal follow-up studies.

In addition, combined immunosuppressive regimens used in other gene-related forms of LDs might offer insights. In studies of colony-stimulating factor 1 receptor *(CSF1R)*-related adult-onset leukoencephalopathy with axonal spheroids and pigmented glia, one asymptomatic mutation carrier who received long-term combination therapy with immunosuppressive agents, including prednisone, hydroxychloroquine, methotrexate, and adalimumab, showed no white matter abnormalities on brain MRI. This observation suggests that immunosuppressive agents might delay or prevent disease progression. However, the use of immunosuppressive agents in *AARS1*/*AARS2*-related leukodystrophies requires further investigation [107].

### 6.4. Hematopoietic Stem Cell Transplantation and Cell and Gene Therapy

Hematopoietic stem cell transplantation (HSCT) confers a unique therapeutic advantage in disorders in which pathogenic gene mutations lead to the accumulation of toxic substrates within the central nervous system, as systemically administered therapies are largely unable to cross the blood–brain barrier [108]. This advantage is attributed to the capacity of hematopoietic stem and progenitor cells (HSPCs) and myeloid lineage cells to migrate into the CNS and replace resident populations of tissue macrophages and microglia-like cells, thereby enabling sustained correction of disease-relevant cellular dysfunctions [109,110]. In other forms of LDs, HSCT was shown to slow neurological deterioration and improve neuroimaging abnormalities in *CSF1R*-related adult-onset leukoencephalopathy with axonal spheroids and pigmented glia, with some patients maintaining stable cognitive and gait function [111,112,113,114]. Recently, microglial replacement achieved through bone marrow transplantation corrected functional deficits caused by *CSF1R* mutations [115]. In X-linked adrenoleukodystrophy (X-ALD; OMIM: 300100), gene-modified HSCT halted the progression of white matter demyelination and stabilized neurological function by reconstituting functional myeloid cells, including microglia [116]. Subsequently, multicenter phase II-III clinical trials demonstrated that 88% of 17 ALD patients were alive without major functional disability, treatment-related severe toxicity, or graft-versus-host disease at 24 months [117], supporting the therapeutic potential of HSCT for LDs. However, there are no reports of HSCT treatment in *AARS1*/*AARS2*-related leukodystrophies to date. The efficacy and safety of HSCT in this disease requires further clinical investigation.

### 6.5. Targeted Drug Therapy and Enzyme Replacement

Targeted drug therapies have been explored as potential therapeutic strategies in certain forms of LDs by modulating disease-relevant signaling pathways. For example, an ongoing clinical trial evaluated targeted agents for *CSF1R*-related adult-onset leukoencephalopathy with axonal spheroids and pigmented glia (ClinicalTrials.gov Identifier: NCT05677659), which might provide valuable insights for therapeutic development in LDs. Nevertheless, targeted pharmacological interventions directly addressing the molecular defects of *AARS1*/*AARS2*-related leukodystrophies remained to be explored.

Enzyme replacement therapy (ERT), in contrast, has been successfully applied in lysosomal storage LDs characterized by enzyme deficiency and toxic substrate accumulation within the central nervous system, such as metachromatic leukodystrophy (MLD; OMIM: 250100) [118]. However, *AARS1*/*AARS2*-related leukodystrophies are not associated with lysosomal enzyme deficiency or degradable substrate accumulation, but are instead driven by defects in cytosolic or mitochondrial protein translation and energy metabolism. Accordingly, ERT is considered biologically unsuitable for *AARS1*/*AARS2*-related leukodystrophies, and no clinical or preclinical evidence supports its application to date.

### 6.6. Genetic Counseling and Prevention

Systematic genetic counseling and prenatal genetic testing plays an important role in the long-term prevention and control of *AARS1*/*AARS2*-related leukodystrophies [106]. Once pathogenic variants in *AARS1* or *AARS2* are identified in an affected family member, carrier testing for at-risk relatives as well as prenatal or preimplantation genetic testing is feasible.

## 7. Discussion

*AARS1*/*AARS2*-related leukodystrophies are caused by pathogenic mutations in alanyl-tRNA synthetase genes, constituting a rare but informative group of inherited white matter disorders. Accumulating clinical, genetic, and experimental evidence indicates that disruption of the canonical aminoacylation and editing functions of AARS1 and AARS2 proteins represents a central pathogenic mechanism. Such disruption impairs translational fidelity and induces cellular stress responses. Pathogenic variants in *AARS2* dysfunction also compromises mitochondrial translation and bioenergetics. The basis of selective white matter injury remains unclear.

In addition to canonical translational defects, recent studies reveal non-canonical functions of AARS1 and AARS2 proteins. Similar aminoacylation or editing defects and bioenergetic dysfunction occur in other aaRS-related disorders. Their downstream effects, however, depend on the affected enzyme and variant. For example, aberrant protein interactions occur in both *AARS1*- and *GARS1*-related peripheral neuropathies. Their molecular consequences differ. Protein lysine lactylation is a distinctive non-canonical activity of AARS1/AARS2 among the human aaRSs examined to date. Its contribution to leukodystrophy remains unproven.

Experimental models reproduce selected features of AARS1/AARS2 Protein dysfunction. These include translational infidelity, proteotoxic stress, and mitochondrial abnormalities. However, the models do not fully recapitulate the characteristic white matter pathology. Studies using patient-derived neural cells remain limited. The effects on oligodendrocyte development and myelination require further investigation.

The marked clinical heterogeneity observed among *AARS1*/*AARS2*-related disorders highlights the complexity of genotype–phenotype relationships. The clinical spectrum includes leukodystrophy, peripheral neuropathy, developmental and epileptic encephalopathy, cardiomyopathy, and ovarian failure. Disease manifestations may be influenced by variant location, residual aminoacylation or editing activity, and tissue-specific vulnerability. However, the number of reported cases remains limited, and functional characterization is incomplete. These limitations preclude robust genotype–phenotype correlations.

Therapeutic options for *AARS1*/*AARS2*-related leukodystrophies are limited to symptomatic and supportive management. No disease-modifying treatments are available. Therapeutic advances in other LDs may provide useful perspectives. However, their application in *AARS1*/*AARS2*-related leukodystrophies requires careful evaluation. Integration of clinical phenotyping with functional characterization of variants is essential for understanding pathomechanisms and developing therapeutic strategies.

## Data Availability

The original contributions presented in this study are included in the article and Supplementary Materials. Further inquiries can be directed to the corresponding author.

## Supplementary Materials

The following supporting information can be downloaded at: https://www.mdpi.com/article/10.3390/genes17080872/s1, Table S1: Reported *AARS1* and *AARS2* variants included in the mutation spectrum analysis; Table S2: Clinical features and reported outcomes of *AARS1*- and *AARS2*-related leukodystrophy cases.

## Abbreviations

The following abbreviations are used in this manuscript:

aaRS	Aminoacyl-tRNA synthetase
AARS1	Alanyl-tRNA synthetase 1
AARS2	Alanyl-tRNA synthetase 2
AD	Autosomal dominant inheritance
ADLD	Adult-onset autosomal dominant leukodystrophy
AlaRS	Alanyl-tRNA synthetase
AlaXp	Alanyl-tRNA synthetase domain containing 1
ALSP	Adult-onset leukoencephalopathy with axonal spheroids and pigmented glia
ALXDRD	Alexander disease
APP	Amyloid precursor protein
AR	Autosomal recessive inheritance
cGAS	cyclic GMP-AMP synthase
CMT2N	Charcot–Marie–Tooth disease, type 2N
COXPD8	Combined oxidative phosphorylation deficiency type 8
CPT2	Carnitine palmitoyltransferase 2
CT	Computed tomography
DARS2	Aspartyl-tRNA synthetase 2
DEE29	Developmental and epileptic encephalopathy type 29
DWI	Diffusion-weighted imaging
GLD	Globoid cell leukodystrophy
HSCT	Hematopoietic stem cell transplantation
LD	Leukodystrophy
LKENP	Progressive leukoencephalopathy with ovarian failure
LLPS	liquid–liquid phase separation
MLD	Metachromatic leukodystrophy
mTORC1	Mechanistic target of rapamycin complex 1
OXPHOS	Oxidative phosphorylation
PDHA1	Pyruvate dehydrogenase E1 component alpha subunit
PKM2	Pyruvate kinase M2
POI	Premature ovarian insufficiency
PPARγ	Peroxisome proliferator-activated receptor γ
TTD8	Nonphotosensitive trichothiodystrophy type 8
X-ALD	X-linked adrenoleukodystrophy
XLR	X-linked recessive inheritance
