# Supplementary material for: Advances in Research on AARS1/AARS2-Related Disorders: A Focus on Leukodystrophies
Source: Genes (Basel). 2026 Jul 26;17(8):872. doi: 10.3390/genes17080872 (PMC13512900; doi:10.3390/genes17080872)
Supplement: Supplementary file 1 [file genes-17-00872-s001.zip › genes-4404150-supplementary.pdf]

Table S1. Reported *AARS1* and *AARS2* variants included in the mutation spectrum analysis

Phenotype	Record No.	Nucleotide change (HGVS)	Protein change (HGVS)	Variant type	Inheritance	Zygosity	Reference
AARS1-LD	V1	c.455G>T	p.Cys152Phe	Missense	AD	Het	[6]
AARS1-LD	V2	c.455G>T	p.Cys152Phe	Missense	AD	Het	[6]
AARS1-LD	V3	c.296A>G<br>c.778A>G	p.Glu99Gly<br>p.Thr260Ala	Missense<br>Missense	AR	Comp Het	[13]
AARS1-LD	V4	c.562_563delinsCA<br>c.1574G>A	p.Ser188His<br>p.Cys525Tyr	Missense<br>Missense	AR	Comp Het	[13]
AARS1-LD	V5	c.1741G>A<br>c.1741G>A	p.Gly581Ser<br>p.Gly581Ser	Missense<br>Missense	AR	Hom	[13]
AARS1-LD	V6	c.1997T>C<br>exon1-4 Deletion	p.Val666Ala<br>p.?	Missense<br>Multi-exon deletion	AR	Comp Het	[13]
AARS1-LD	V7	c.1817C>T<br>c.1817C>T	p.Thr606Ile<br>p.Thr606Ile	Missense<br>Missense	AR	Hom	[14]
AARS1-CMT2N	V8	c.986G>A	p.Arg329His	Missense	AD	Het	[34]
AARS1-CMT2N	V9	c.976C>T	p.Arg326Trp	Missense	AD	Het	[50]
AARS1-CMT2N	V10	c.1880C > T	p.Ser627Leu	Missense	AD	Het	[15]
AARS1-CMT2N	V11	c.1009G>A	p.Glu337Lys	Missense	AD	Het	[15]
AARS1-CMT2N	V12	c.211A>T	p.Asn71Tyr	Missense	AD	Het	[52]
AARS1-CMT2N	V13	c.304G>C	p.Gly102Arg	Missense	AD	Het	[53]
AARS1-CMT2N	V14	c.2063A>G	p.Glu688Gly	Missense	AD	Het	[31]
AARS1-CMT2N	V15	c.2677G>A	p.Asp893Asn	Missense	AD	Het	[51]
AARS2-LD	V16	c.149T>G<br>c.1561C>T	p.Phe50Cys<br>p.Arg521*	Missense<br>Nonsense	AR	Comp Het	[7]

Table S1. (continued). Reported *AARS1* and *AARS2* variants included in the mutation spectrum analysis

Phenotype	Record No.	Nucleotide change (HGVS)	Protein change (HGVS)	Variant type	Inheritance	Zygosity	Reference
AARS2-LD	V17	c.2893G>A<br>c.1213G>A	p.Gly965Arg<br>p.Glu405Lys	Missense<br>Missense	AR	Comp Het	[7]
AARS2-LD	V18	c.1609C>T;<br>c.2350del<br>c.595C>T;<br>c.2188G>A	p.Gln537*<br>p.Glu784Serfs*9<br>p.Arg199Cys<br>p.Val730Met	Nonsense<br>Frameshift<br>Missense<br>Missense	AR	Comp Het	[7]
AARS2-LD	V19	c.230C>T<br>c.595C>T<br>c.2188G>A	p.Ala77Val<br>p.Arg199Cys<br>p.Val730Met	Missense<br>Missense<br>Missense	AR	Comp Het	[7]
AARS2-LD	V20	c.595C>T;<br>c.2188G>A<br>c.390_392del	p.Arg199Cys<br>p.val730Met<br>p.Phe131del	Missense<br>Missense<br>In-frame deletion	AR	Comp Het	[7]
AARS2-LD	V21	c.595C>T<br>c.2188G>A<br>c.2611dup	p.Arg199Cys<br>p.val730Met<br>p.Thr871Asnfs*21	Missense<br>Missense<br>Frameshift	AR	Comp Het	[7]
AARS2-LD	V22	c.578T>G<br>c.595C>T	p.Leu193*<br>p.Arg199Cys	Nonsense<br>Missense	AR	Comp Het	[59]
AARS2-LD	V23	c.1145C>A<br>c.2255+1G>A	p.Thr382Lys<br>p.?	Missense<br>Splice-site	AR	Comp Het	[60]
AARS2-LD	V24	c.1519G>C<br>c.2165G>A	p.Val507Leu<br>p.Arg722Gln	Missense<br>Missense	AR	Comp Het	[61]
AARS2-LD	V25	c.2265dupA<br>c.650C>T	p.Arg756fs<br>p.Pro217Leu	Frameshift<br>Missense	AR	Comp Het	[62]
AARS2-LD	V26	c.595C>T<br>c.390_392del	p.Arg199Cys<br>p.Phe131del	Missense<br>In-frame deletion	AR	Comp Het	[63]

Table S1. (continued). Reported *AARS1* and *AARS2* variants included in the mutation spectrum analysis

Phenotype	Record No.	Nucleotide change (HGVS)	Protein change (HGVS)	Variant type	Inheritance	Zygosity	Reference
AARS2-LD	V27	c.595C>T<br>c.236T>A	p.Arg199Cys<br>p.Met79Lys	Missense<br>Missense	AR	Comp Het	[63]
AARS2-LD	V28	c.595C>T<br>c.2611_2612insA	p.Arg199Cys<br>p.Thr871Asnfs	Missense<br>Frameshift	AR	Comp Het	[63]
AARS2-LD	V29	c.179C>A<br>c.1703_1704del	p.Pro60His<br>p.Gln568fs	Missense<br>Frameshift	AR	Comp Het	[64]
AARS2-LD	V30	c.595C>T<br>c.2557C>T	p.Arg199Cys<br>p.Arg853Trp	Missense<br>Missense	AR	Comp Het	[65]
AARS2-LD	V31	c.595C>T<br>Deletion of exons 5–7	p.Arg199Cys<br>p.?	Missense<br>Multi-exon deletion	AR	Comp Het	[65]
AARS2-LD	V32	c.965G>A<br>c.334 G>C	p.Arg322His<br>p.Gly112Arg	Missense<br>Missense	AR	Comp Het	[66]
AARS2-LD	V33	c.130G>C<br>c.130G>C	p.Ala44Pro<br>p.Ala44Pro	Missense<br>Missense	AR	Hom	[67]
AARS2-LD	V34	c.452T>C<br>c.452T>C	p.Met151Thr<br>p.Met151Thr	Missense<br>Missense	AR	Hom	[68]
AARS2-LD	V35	c.650C>T<br>c.650C>T	p.Pro217Leu<br>p.Pro217Leu	Missense<br>Missense	AR	Hom	[69]
AARS2-LD	V36	c.647dupG<br>c.595C>T	p.C218LfsX6<br>p.Arg199Cys	Frameshift<br>Missense	AR	Het; Het	[70]
AARS2-LD	V37	c.718C>T<br>c.1040+1G>A	p.Leu240Phe<br>p.?	Missense<br>Splice-site	AR	Comp Het	[71]
AARS2-LD	V38	c.452T>C<br>c.2557C>T	p.Met151Thr<br>p.Arg853Try	Missense<br>Missense	AR	Comp Het	[23]

Table S1. (continued). Reported *AARS1* and *AARS2* variants included in the mutation spectrum analysis

Phenotype	Record No.	Nucleotide change (HGVS)	Protein change (HGVS)	Variant type	Inheritance	Zygosity	Reference
AARS2-LD	V39	c.1874G > A<br>c.179C > A	p.Arg625His<br>p.Pro60His	Missense<br>Missense	AR	Het; Het	[72]
AARS2-LD	V40	c.2864G>A<br>c.1036C>A	p.Trp955*<br>p.Pro346fs*18	Nonsense<br>Frameshift	AR	Het; Het	[73]
AARS2-LD	V41	c.452T>C<br>c.2146–2A>G	p.Met151Thr<br>p.?	Missense<br>Splice-site	AR	Comp Het	[74]
AARS2-LD	V42	c.452T>C<br>c.452T>C	p.Met151Thr<br>p.Met151Thr	Missense<br>Missense	AR	Hom	[58]
AARS2-LD	V43	c.452T>C<br>c.1871G>A	p.Met151Thr<br>p.Trp624*	Missense<br>Nonsense	AR	Het; Het	[58]
AARS2-LD	V44	c.637G>C<br>c.965G>A	p.Gly213Arg<br>p.Arg322His	Missense<br>Missense	AR	Het; Het	[58]
AARS2-LD	V45	c.802A>G<br>c.1871G>A	p.Met268Val<br>p.Trp624*	Missense<br>Nonsense	AR	Het; Het	[58]
AARS2-LD	V46	c.179C>A<br>c.2752C>T	p.Pro60His<br>p.Gln918*	Missense<br>Nonsense	AR	Comp Het	[75]
AARS2-LD	V47	c.452T>C<br>c.963C>A	p.Met151Thr<br>p.Tyr321*	Missense<br>Nonsense	AR	Comp Het	[76]
AARS2-LD	V48	c.452T>C<br>c.963C>A	p.Met151Thr<br>p.Tyr321*	Missense<br>Nonsense	AR	Comp Het	[76]
AARS2-LD	V49	c.452T>C<br>c.737T>A	p.Met151Thr<br>p.Met246Lys	Missense<br>Missense	AR	Comp Het	[77]
AARS2-LD	V50	c.1871G>A<br>c.452T>C	p.Trp624*<br>p.Met151Thr	Nonsense<br>Missense	AR	Comp Het	[57]

Table S1. (continued). Reported *AARS1* and *AARS2* variants included in the mutation spectrum analysis

Phenotype	Record No.	Nucleotide change (HGVS)	Protein change (HGVS)	Variant type	Inheritance	Zygosity	Reference
AARS2-LD	V51	c.1871G>A<br>c.452T>C	p.Trp624*<br>p.Met151Thr	Nonsense<br>Missense	AR	Comp Het	[57]
AARS2-LD	V52	c.802A>G<br>c.1871G>A	p.Met268Val<br>p.Trp624*	Nonsense<br>Missense	AR	Comp Het	[57]
AARS2-LD	V53	c.179C>A<br>c.1703_1704del	p.Pro60His<br>p.Gln568fs	Missense<br>Frameshift	AR	Comp Het	[57]
AARS2-LD	V54	c.452T>C<br>c.452T>C	p.Met151Thr<br>p.Met151Thr	Missense<br>Missense	AR	Hom	[57]
AARS2-LD	V55	c.1871G>A<br>c.452T>C	p.Trp624*<br>p.Met151Thr	Nonsense<br>Missense	AR	Comp Het	[78]
AARS2-LD	V56	c.2557C>T<br>c.595C>T	p.Arg853Trp<br>p.Arg199Cys	Missense<br>Missense	AR	Comp Het	[79]
AARS2-LD	V57	c.2358_2364+7del<br>c.1709delG	p.?<br>p.Gly570Alafs*21	Splice-site<br>Frameshift	AR	Comp Het	[80]
AARS2-LD	V58	c.1041–1G>A<br>c.595C>T	p.?<br>p.Arg199Cys	Splice-site<br>Missense	AR	Comp Het	[24]
AARS2-LD	V59	c.1188G>A<br>c.1709delG	p.?<br>p.Gly570Alafs*21	Splice-site<br>Frameshift	AR	Comp Het	[24]
AARS2-LD	V60	c.1188G>A<br>c.1709delG	p.?<br>p.Gly570Alafs*21	Splice-site<br>Frameshift	AR	Comp Het	[24]
AARS2-LD	V61	c.892_894del<br>c.2234_2235del	p.Gln298del<br>p.Ser745Cysfs*60	In-frame deletion<br>Frameshift	AR	Comp Het	[24]
AARS2-LD	V62	c.595C>T<br>c.595C>T	p.Arg199Cys<br>p.Arg199Cys	Missense<br>Missense	AR	Hom	[24]

Table S1. (continued). Reported *AARS1* and *AARS2* variants included in the mutation spectrum analysis

Phenotype	Record No.	Nucleotide change (HGVS)	Protein change (HGVS)	Variant type	Inheritance	Zygosity	Reference
AARS2-LD	V63	c.1691T>C<br>c.179C>A	p.Phe564Ser<br>p.Pro60His	Missense<br>Missense	AR	Comp Het	[25]
AARS2-LD	V64	c.985C>T<br>c.452T>C	p.Arg329Cys<br>p.Met151Thr	Missense<br>Missense	AR	Comp Het	[81]
AARS2-LD	V65	c.965G> A<br>c.179C> A	p.Arg322His<br>p.Pro60His	Missense<br>Missense	AR	Comp Het	[82]
AARS2-LD	V66	c.718C>T<br>c.1040+1G>A	p.Leu240Phe<br>p.?	Missense<br>Splice-site	AR	Comp Het	[83]
AARS2-LD	V67	c.1699G>T<br>c.466C>G	p.Glu567*<br>p.Leu156Val	Nonsense<br>Missense	AR	Comp Het	[84]
AARS2-LD	V68	c.514T>A<br>c.595C>T	p.Phe172Ile<br>p.Arg199Cys	Missense<br>Missense	AR	Comp Het	[84]
AARS2-LD	V69	c. 452T>C<br>c. 701dupC	p.Met151Thr<br>p.?	Missense<br>Frameshift	AR	Comp Het	[85]
AARS2-LD	V70	c. 452T>C<br>c. 244T>A	p.Met151Thr<br>p.Phe82Ile	Missense<br>Missense	AR	Comp Het	[86]

Note: For *AARS1*-LD and *AARS2*-LD, variants are listed on a per-case basis, reflecting individual patients reported in the literature. For *AARS1*-CMT2N, variants are listed once, irrespective of the number of affected individuals, families, or publications; all relevant references are provided. Variants identified during screening but not established as disease-associated in the original publications were excluded. This table is intended to document the literature sources for variants included in the mutation spectrum analysis and is not intended for quantitative comparison across phenotypes. 'Het; Het' indicates that both variants were heterozygous, but their phase was not established because parental testing was not performed. Abbreviations: LD, leukodystrophy; CMT2N, Charcot-Marie-Tooth disease type 2N; HGVS, Human Genome Variation Society; AD, autosomal dominant; AR, autosomal recessive; Het, heterozygous; Comp Het, compound heterozygous; Hom, homozygous.

Table S2. Clinical features and reported outcomes of *AARS1*- and *AARS2*-related leukodystrophy cases

Phenotype	Record No. (S1)	Sex	Age at Onset[1]	Major Clinical Features	Reported Outcome	Reference
AARS1-LD	V1	Male	46Y	Personality change, frontal syndrome, pyramidal and deep sensory signs, hyperactivity, hemianopia, and generalized rigidity	Deceased at 57 years	[6]
AARS1-LD	V2	Female	34Y	Cognitive impairment, deep sensory ataxia, dystonic and ballistic movements, gait impairment, pyramidal signs, and visual field loss	Deceased; age NR	[6]
AARS1-LD	V3	Female	27Y	Peripheral neuropathy, progressive lower-limb spasticity, gait imbalance, cognitive decline, visual impairment, and dysarthria	Not reported	[13]
AARS1-LD	V4	Female	12M	Developmental regression, spastic tetraparesis, focal epilepsy, optic atrophy	Severe neurological impairment; stable at 12 years	[13]
AARS1-LD	V5	Female	36M	Developmental regression, spastic tetraparesis, dystonia, focal epilepsy, microcephaly	Deceased at 19 years	[13]
AARS1-LD	V6	Male	12Y	Right-sided hemiparesis progressing to tetraparesis, motor and cognitive regression, bradykinesia, dystonic movements, and severe spastic dysarthria	Not reported	[13]
AARS1-LD	V7	Male	23Y	Gait difficulty, progressive dysarthria, blurred vision, cognitive decline, hypertonia, hyperreflexia, decreased muscle strength	Wheelchair-dependent at 25 years	[14]
AARS2-LD	V16	Female	2Y	Developmental delay; cerebellar ataxia, tremor, cognitive deterioration, psychosis, and ovarian failure	Walk with support at 30 years	[7]

[1] Age at onset was defined as the age at which the first disease-related symptom occurred

Table S2. (continued). Clinical features and reported outcomes of *AARS1*- and *AARS2*-related leukodystrophy cases

Phenotype	Record No. (S1)	Sex	Age at Onset	Major Clinical Features	Reported Outcome	Reference
AARS2-LD	V17	Male	Infancy	Congenital nystagmus; learning difficulties, hemiparesis, ataxia, dystonia, spasticity, dysarthria, cognitive decline, and dysphagia	Not reported	[7]
AARS2-LD	V18	Female	28Y	Ovarian failure; depression, cognitive deterioration, frontal behavioral dysfunction, urinary incontinence, apraxia, and seizures	Bedridden at 36 years	[7]
AARS2-LD	V19	Female	23Y	Ovarian failure; tremor, rapid cognitive and behavioral deterioration, dystonia, cerebellar ataxia, and spasticity	Died at 28 years	[7]
AARS2-LD	V20	Female	40Y	Ovarian failure; depression and rapid cognitive deterioration	Died at 46 years	[7]
AARS2-LD	V21	Female	20Y	Ovarian failure; gait impairment, depression, spastic paraparesis, and ataxia	Wheelchair-bound at 25 years	[7]
AARS2-LD	V22	Male	18Y	Behavioral changes, mania, and paranoid delusions, cognitive decline, nystagmus, dysarthria, dysphagia, and extrapyramidal and pyramidal signs	Bedridden at 29 years	[59]
AARS2-LD	V23	Female	30Y	Behavioral changes and cognitive decline, followed by left-sided limb dysfunction, urinary incontinence, secondary amenorrhea, frontal dysfunction, apraxia, hyperreflexia, and rigidity	Bedridden at 38 years	[60]

Table S2. (continued). Clinical features and reported outcomes of *AARS1*- and *AARS2*-related leukodystrophy cases

Phenotype	Record No. (S1)	Sex	Age at Onset	Major Clinical Features	Reported Outcome	Reference
AARS2-LD	V24	Male	18M	Progressive visual impairment, bilateral optic atrophy, pigmentary retinopathy, and motor-predominant demyelinating polyneuropathy	Not reported	[61]
AARS2-LD	V25	Male	17Y	Progressive limb tremor, weakness, nystagmus, anarthria, ataxia, gait impairment, cognitive decline, and spastic tetraparesis	Dependent at 8-month follow-up	[62]
AARS2-LD	V26	Female	Not reported	Mild cognitive delay and primary amenorrhea; gait disturbance and cognitive deterioration from 44 years	Not reported	[63]
AARS2-LD	V27	Female	32Y	Secondary amenorrhea, depression, and cognitive decline, followed by progressive spastic tetraparesis	Died at 36 years	[63]
AARS2-LD	V28	Female	6Y	Downbeat and gaze-evoked nystagmus from 6 years; primary amenorrhea; progressive gait disturbance, spastic paraparesis, and right cerebellar syndrome from 23 years, with preserved cognition	Not reported	[63]
AARS2-LD	V29	Male	29Y	Progressive right lower-limb weakness, gait instability, spastic gait, dysarthria, dysphagia, aphasia, and severe cerebellar ataxia	Rapid deterioration within 1 year	[64]

Table S2. (continued). Clinical features and reported outcomes of *AARS1*- and *AARS2*-related leukodystrophy cases

Phenotype	Record No. (S1)	Sex	Age at Onset	Major Clinical Features	Reported Outcome	Reference
AARS2-LD	V30	Male	25Y	Hand tremor followed by progressive cognitive, psychiatric, and motor decline, urinary incontinence, gait instability, anarthria, immobility, and dysphagia	Nonverbal, Nonambulatory at 48 years	[65]
AARS2-LD	V31	Female	7Y	Nonverbal, wheelchair-dependent, and nonambulatory at 48 years	Not reported	[65]
AARS2-LD	V32	Male	20Y	Progressive gait ataxia, bilateral lower-limb stiffness, cognitive decline, abnormal behavior, nystagmus, and asymmetric spastic paraparesis	Not reported	[66]
AARS2-LD	V33	Male	20Y	Dysphagia, dysarthria, gait clumsiness, spastic paraparesis, pyramidal and pseudobulbar signs, and mild frontal executive dysfunction	Not reported	[67]
AARS2-LD	V34	Male	40Y	Gait difficulty, cognitive decline, behavioral abnormality, cerebellar ataxia, and generalized hyperreflexia	No significant cognitive improvement	[68]
AARS2-LD	V35	Male	27Y	Increasingly unresponsive and unable to walk unaided at 19-month follow-up	Unable to walk unaided at 19-month	[69]
AARS2-LD	V36	Male	13Y	Behavioral change, psychosis, postural and kinetic tremor, parkinsonism, nystagmus, and mild ataxia	Not reported	[70]
AARS2-LD	V37	Female	22Y	Premature ovarian failure, progressive lower-limb weakness, cognitive decline, behavioral changes, tremor, bradykinesia, rigidity, and ataxia	Bedbound at follow-up	[71]

Table S2. (continued). Clinical features and reported outcomes of *AARS1*- and *AARS2*-related leukodystrophy cases

Phenotype	Record No. (S1)	Sex	Age at Onset	Major Clinical Features	Reported Outcome	Reference
AARS2-LD	V38	Male	20Y	Progressive lower-limb weakness, dysarthria, dysphagia, cerebellar ataxia, parkinsonism, and cognitive decline	Bedridden at 9-month	[23]
AARS2-LD	V39	Male	Not reported	Gait apraxia with compensatory dance-like upper-limb movements, dysarthria, mild spasticity, hyperreflexia, and mild cognitive impairment	Not reported	[72]
AARS2-LD	V40	Female	15Y	Primary amenorrhea, uterus infantilis, rapidly progressive cognitive and behavioral decline, pyramidal signs, psychosis, and anosognosia	Loss of autonomy at 43 years	[73]
AARS2-LD	V41	Male	28Y	Cognitive decline, personality and behavioral changes, unsteady gait, horizontal nystagmus, and bladder and bowel dysfunction	Not reported	[74]
AARS2-LD	V42	Male	37Y	Cognitive impairment and fatigability	Not reported	[58]
AARS2-LD	V43	Male	28Y	Spastic hemiplegia, cognitive impairment, psychiatric symptoms, nystagmus, dysarthria, and tremor	Not reported	[58]
AARS2-LD	V44	Female	35Y	Premature ovarian failure, cognitive impairment, psychiatric changes, and involuntary movements	Not reported	[58]
AARS2-LD	V45	Male	3M	Delayed motor development, cognitive impairment, psychiatric symptoms, ataxia, pyramidal signs, tremor, nystagmus, and dysarthria	Not reported	[58]

Table S2. (continued). Clinical features and reported outcomes of *AARS1*- and *AARS2*-related leukodystrophy cases

Phenotype	Record No. (S1)	Sex	Age at Onset	Major Clinical Features	Reported Outcome	Reference
AARS2-LD	V46	Female	39Y	Progressive cognitive decline, psychiatric and behavioral abnormalities, urinary and fecal incontinence, ataxia, spastic gait, and ovarian failure	Died at 45 years	[75]
AARS2-LD	V47	Female	33Y	Primary ovarian failure, progressive cognitive impairment, bradykinesia, postural tremor, and spastic-ataxic gait	Wheelchair-bound at 39 years	[76]
AARS2-LD	V48	Male	35Y	Cognitive impairment, left-leg dystonia, urinary incontinence, and spastic-ataxic gait	Required walking assistance at 37 years	[76]
AARS2-LD	V49	Male	28Y	Progressive dysarthria, gait disturbance, right-sided weakness, spasticity, and ankle clonus	Not reported	[77]
AARS2-LD	V50	Female	22Y	Right-hand tremor, spasticity, dystonia, dysarthria, cognitive decline, and ovarian failure	Not reported	[57]
AARS2-LD	V51	Male	12Y	Cognitive decline, right-hand tremor, dysarthria, nystagmus, spasticity, and gait impairment	Not reported	[57]
AARS2-LD	V52	Male	3M	Developmental delay, cognitive and behavioral changes, dysarthria, dysphagia, spasticity, ataxia, tremor, and nystagmus	Not reported	[57]
AARS2-LD	V53	Male	29Y	Memory impairment, personality changes, dysarthria, dysphagia, urinary incontinence, and spastic gait	Not reported	[57]

Table S2. (continued). Clinical features and reported outcomes of *AARS1*- and *AARS2*-related leukodystrophy cases

Phenotype	Record No. (S1)	Sex	Age at Onset	Major Clinical Features	Reported Outcome	Reference
AARS2-LD	V54	Male	Not reported	Personality changes, cognitive impairment, nystagmus, dysarthria, and lower-limb spasticity	Not reported	[57]
AARS2-LD	V55	Male	27Y	Tremor, progressive limb weakness, gait instability, cognitive decline, dysarthria, spasticity, and urinary and fecal incontinence	Bedridden	[78]
AARS2-LD	V56	Female	28Y	Ovarian failure, progressive cognitive decline, limb weakness, spasticity, dysphagia, and pyramidal signs	Not reported	[79]
AARS2-LD	V57	Female	17Y	Secondary amenorrhea, depression, progressive cognitive decline, spasticity, gait impairment, and epilepsy	Not reported	[80]
AARS2-LD	V58	Female	Mid-20s	Premature ovarian failure, calculation, anxiety, ataxia, asymmetric spastic paraparesis, and left upper-limb apraxia	Not reported	[24]
AARS2-LD	V59	Male	Late 30s	Neuropsychiatric and behavioral changes, executive dysfunction, memory impairment, and upper-limb tremor	Required residential care within 1 year of onset	[24]
AARS2-LD	V60	Male	Mid-20s	Progressive speech and swallowing difficulties and rapid motor decline	Died within 1 year of onset	[24]
AARS2-LD	V61	Male	Mid-adolescence	Psychiatric and behavioral changes, ataxic gait, visual disturbance, psychosis, spastic paraparesis, bradykinesia, and hand dystonia	Died in late adolescence	[24]

Table S2. (continued). Clinical features and reported outcomes of *AARS1*- and *AARS2*-related leukodystrophy cases

Phenotype	Record No. (S1)	Sex	Age at Onset	Major Clinical Features	Reported Outcome	Reference
AARS2-LD	V62	Male	Mid-40s	Dystonia, dysarthria, cognitive decline, neuropsychiatric symptoms, pseudobulbar palsy, spastic quadriparesis, and parkinsonism	Died 1 year after symptom onset	[24]
AARS2-LD	V63	Female	32Y	Right-sided limb clumsiness, dysarthria, cognitive impairment, rigid spasticity, dysphagia, incontinence, and behavioral changes	Not reported	[25]
AARS2-LD	V64	Male	13Y	Progressive gait instability, lower-limb spasticity and weakness, ataxia, horizontal nystagmus, and hyperreflexia	Not reported	[81]
AARS2-LD	V65	Male	33Y	Epileptic seizures, progressive cognitive decline, personality and behavioral changes, mixed aphasia, hypertonia, loss of independent ambulation, and urinary and fecal incontinence	Not reported	[82]
AARS2-LD	V66	Female	22Y	Secondary amenorrhea, lower-limb weakness, gait instability, cognitive impairment, and dysarthria	Lower-limb weakness worsened over 12 months	[83]
AARS2-LD	V67	Female	>35Y	Mild axial and limb ataxic syndrome and early cognitive decline	Not reported	[84]
AARS2-LD	V68	Male	16Y	Cognitive impairment and mild limb ataxia	Died about 2 years later	[84]

Table S2. (continued). Clinical features and reported outcomes of *AARS1*- and *AARS2*-related leukodystrophy cases

Phenotype	Record No. (S1)	Sex	Age at Onset	Major Clinical Features	Reported Outcome	Reference
AARS2-LD	V69	Male	16Y	Limb spasticity and incoordination, horizontal nystagmus, scanning speech, bilateral foot drop, action tremor, and demyelinating motor polyneuropathy	Stable during 36 months of intravenous immunoglobulin treatment	[85]
AARS2-LD	V70	Male	15Y	Progressive limb tremor, cerebellar ataxia, mild dysarthria, horizontal and vertical nystagmus, hypotonia, and hyperreflexia	Not reported	[86]

Note: Record No. (S1) corresponds to the record number in Table S1. Reported Outcome reflects the latest clinical status explicitly documented in the original publication and does not indicate the patient's current status; 'Not reported' indicates that the corresponding information was not provided. Y, years; M, months
